# Development of a Colloidal Gold-Based Immunochromatographic Strip for Rapid Detection of Severe Acute Respiratory Syndrome Coronavirus 2 Spike Protein

**DOI:** 10.3389/fimmu.2021.635677

**Published:** 2021-03-11

**Authors:** Ge Li, Aiping Wang, Yumei Chen, Yaning Sun, Yongkun Du, Xun Wang, Peiyang Ding, Rui Jia, Yanwei Wang, Gaiping Zhang

**Affiliations:** ^1^College of Veterinary Medicine, Henan Agricultural University, Zhengzhou, China; ^2^School of Life Sciences, Zhengzhou University, Zhengzhou, China; ^3^Henan Zhongze Biological Engineering Co., Zhengzhou, China

**Keywords:** SARS-CoV-2, receptor binding domain, monoclonal antibody, gold immunochromatographic strip, rapid detection

## Abstract

The outbreak and worldwide pandemic of the novel severe acute respiratory syndrome coronavirus 2 (SARS-CoV-2) have a significant impact on global economy and human health. In order to reduce the disease spread, 16 monoclonal antibodies (McAbs) again SARS-CoV-2 were generated by immunized mice with the spike protein receptor binding domain (RBD), which was expressed in Chinese hamster ovary cell (CHO). A colloidal gold-based immunochromatographic strip was developed with two McAbs to detect SARS-CoV-2 spike protein, which can play a potential role in monitoring vaccine quality. The strip is highly specific, detecting only SARS-CoV-2 spike protein, and does not show any non-specific reactions with syndrome coronavirus (SARS-CoV), Middle East respiratory syndrome coronavirus (MERS-CoV) and other coronavirus and influenza viruses. The strip detected subunit vaccine in our laboratory with a detection limit of spike protein of 62.5 ng/mL. This strip provides an effective method in monitoring vaccine quality by detecting the antigen content of spike protein.

## Introduction

The coronavirus disease 2019 (COVID-19) caused by the novel severe acute respiratory syndrome coronavirus 2 (SARS-CoV-2) infection is spreading to more than 200 countries/regions with 50 million infections and 1,263,844 deaths (as of Nov. 12) according to statistics from the World Health Organization (1). Rapid antigen detection (RAD) tests plays an important role in reducing the disease spread (2). On the one hand, RAD tests has the potential to be an important tool for early diagnosis of SARS-CoV-2, especially in cases where molecular methods are limited (3). On the other hand, RAD can monitor the amount of SARS-CoV-2 spike protein available in vaccines. Although RT-PCR and Viral culture are the current gold standards in the diagnosis of SARS-CoV-2 infection, several factors such as skilled staff, requirement of special equipment and price of the reagent limit the use of these time-consuming molecular techniques (4). Hance, researches of different immunochromatographic strips for COVID-19 detection are encouraged (5).

SARS-CoV-2 is a single-stranded positive-stranded RNA virus (6). Its genome encodes four structural proteins: spike protein (S), small protein (E), matrix protein (M) and nucleocapsid protein (N) (7–9). Spike protein is a type I fusion protein that forms trimers on the surface of virus particles. It consists of two subunits, S1 and S2: S1 mainly contains receptor binding domain (RBD), which is responsible for recognizing cell receptors and S2 is responsible for membrane fusion. Angiotensin-converting enzyme 2 (ACE2) is used to enter target cells as a receptor (9–12). Therefore, the RBD of spike protein determines the infectivity of virus and its spread in the host (10, 13, 14). Although immunochromatographic strips developed by some researches use antibodies against SARS-CoV-2 N protein (15). Most of vaccines and antibody therapeutics under development are directed against SARS-CoV-2 spike protein RBD since the protein is the main antigen that induces a protective immune response (16–19).

On April 7, 2020, a large-scale case study published by the CDC in the United States estimated that the Basic Reproduction Number (R0) of SARS-CoV-2 was 5.7 meaning that the transmission capacity of SARS-CoV-2 is much higher than that of SARS-CoV (R0: 0.85–3) (20). The world urgently needs an effective and safe vaccine, which will play a decisive role in global epidemic control. According to the draft candidate vaccines published by the WHO on August 27, 2020, there are 143 vaccines worldwide in preclinical trials, 33 vaccines have entered clinical trials, of which 8 vaccines have entered clinical phase III trials. Recently, vaccines produced in China have been fully marketed. Vaccines under research include widely used traditional vaccines, namely inactivated or attenuated vaccines, genetically engineered recombinant subunit vaccines, adenovirus vector vaccines, recombinant virus vector vaccines, and new vaccines that have not been approved for similar vaccines, mainly including ribose Nucleic acid (RNA) vaccines and deoxyribonucleic acid (DNA) vaccines. Among these vaccines, spike protein-based vaccines occupy a certain number. Currently, the main method of vaccine detection is enzyme-linked immunosorbent assay, but ELISA is cumbersome and prone to non-specific results. It is particularly important that vaccination is beneficial to the prevention and control of the epidemic. A study (21) found that IgG titers persist for more than 4 months after the onset of symptoms, which means that long-term immunity to COVID-19 may be observed or vaccinated. Therefore, it is necessary to develop a gold immunochromatographic strip for rapid detection of SARS-CoV-2 spike protein in order to detect the antigen content of spike protein to monitor vaccine quality.

In this study, monoclonal antibodies (McAbs) against SARS-CoV-2 were generated by immunized mice with the spike protein RBD as an immunogen. A gold immunochromatographic strip specific for SARS-CoV-2 spike protein was then developed using two SARS-CoV-2 specific McAbs, which can detect subunit vaccine in our laboratory with a detection limit of SARS-CoV-2 spike protein of 62.5 ng/mL within 15–30 min.

## Materials and Methods

### Ethics and Biosafety Statements

The experimental research protocol for monoclonal antibody production in mice was approved by the Key Laboratory of Animal Immunology, Henan Academy of Agricultural Sciences, China, in line with its policies and procedures.

### Expression and Purification of Spike Protein RBD

The sequence used for the protein was based on the genomic sequence of the first isolate, Wuhan-Hu-1, which was released on January 10, 2020 (spike protein residues 132–537, GenBank: MN908947.3). The signal peptide sequence of SARS-CoV-2 spike protein RBD (spike protein 1–18 residues) was added to the N terminal of the protein for protein secretion, and a Hexa-His tag was added to the C terminal of the protein for further purification. These constructs were synthesized by Sangon Biotech (Shanghai, China). These constructs were cloned into the pcDNA3.1 (+) vector and transiently transfected into CHO cells when the cell density were 6 × 10^6^ cells/mL. After 3 days, the cells were harvested and centrifuged 10 min at 10,000 × g, 4°C. The supernatant was filtered using a 0.22-μm Stericup filter and purified by Ni affinity chromatography using a HisTrapTM excel 5 mL column (GE Healthcare) in a buffer composed of 20 mM PBS (pH 7.2). The protein size and purity of elution peak were analyzed by SDS-PAGE, and the protein concentration was determined with BCA protein concentration determination kit.

### Generation of Monoclonal Antibodies

Monoclonal antibodies against SARS-CoV-2 were developed following a standard procedure. Briefly, 6-weeks-old female BALB/c mice (*n* = 5) were immunized with the spike protein RBD produced in this study at an immunization dose of 0, 20, 50, 100, and 200 μg each mouse in Freund's adjuvant, respectively. The mice were immunized every 2 weeks. Two weeks after the third immunization, blood was collected to determine the titer of the mouse serum by ELISA. The mouse with the highest titer was selected for super-immunization based on the amount of protein in the first immunization. Cell fusion was performed 3 days after super immunization. Briefly, the spleen of the mouse was aseptically taken to grind and then fused with Sp2/0 myeloma cells at a ratio of 1: 2. The hybridoma cells were screened by ELISA and cloned by the limiting dilution method. The ascitic fluids from the positive hybridomas were produced in mice.

### Preparation of Colloidal Gold and Gold-Labeled Monoclonal Antibodies

Colloidal gold was prepared by trisodium citrate method (22). Briefly, 1 mL of 1% chloroauric acid was added to the erlenmeyer flask with 99 mL double distilled water which was stirring and heating, followed by the rapid addition of 1.6 mL of 1% trisodium citrate solution with rapid stirring. The mixture was boiled for another 5 min and gradually boiled until the color gradually changes from light yellow to deep red and no longer changes in color. The colloidal gold solution was cooled to room temperature and then stored at 4 °C.

McAbs were centrifuged at 12,000 × g for 5 min and incubated with colloidal gold solution for 30 min. Then the 10% bovine serum albumin (BSA) was added to the colloidal gold conjugation and incubated for 10 min. The mixture was then centrifuged at 12,000 × g, 4°C for 30 min to remove any unbound antibody. The pellet was resuspended in boric acid buffer containing 1% BSA.

### Screening of the Strip Paired McAbs by Sandwich Dot-Blot

Among the sixteen positive clones, two McAbs which showing higher binding affinity to SARS-CoV-2 spike protein were selected to establish a rapid detective strip by sandwich Dot-blot. The sandwich Dot-blot was performed as following. Sixteen capture antibodies was blotted on the nitrocellulose membrane (Table 1) at 37°C for 30 min. After blocking the nitrocellulose membrane using phosphate buffered solution (PBS) containing 1% BSA, 200 μL per membrane of sample diluted in antigen dilution buffer were added and then incubated for 30 min. Then the membrane were rinsed five times with PBS containing 0.2% Tween 20. Sixteen colloidal gold conjugated McAbs was added to sixteen membranes with 50 μL every membrane, respectively. The pairing of two specific antibodies were selected by observing the color strength of the nitrocellulose membrane.

**Table 1:** Dot-blot layout of 16 monoclonal antibodies.

**Antibody number**	**a**	**b**	**c**	**d**
1	1F12	2C3	5C8	5E6
2	5E11	6A5	6B7	6C7
3	6E5	8G6	7C7	7H5
4	8C11	9F2	10D3	12F5

### Preparation of the Immunochromatographic Strip

The fiberglass sample pad, conjugate pad, nitrocellulose membrane, and absorpt pad were assembled on the support board sequentially, with 1–2 mm overlapping each other and cut into 2.79-mm pieces (CM 4000 cutter; Bio-Dot) to form an immunochromatographic strip. Briefly, the fiberglass pad was saturated with 10% BSA, and dried at 37°C for 1 h. One SARS-CoV-2 specific McAb was labeled with colloidal gold as conjugated McAb then dispensed on the fiberglass pads to generate conjugate pads. The conjugate pad was dried at 42°C for 50 min. On a 2.79-cm nitrocellulose membrane, the other SARS-CoV-2 specific McAb and the aqueous solution of staphylococal protein A (SPA) were dispensed as test and control lines, respectively. The nitrocellulose membrane was dried at 45°C for 4 h. Pure cellulose fiber was used as an absorbent pad. Immunochromatographic strips were store in a desiccator at 4°C prior to use.

### Specificity Evaluations of the Strip

To evaluate the specificity of the rapid detection strip, the spike protein of coronavirus such as SARS-CoV-2, syndrome coronavirus (SARS-CoV), Middle East respiratory syndrome coronavirus (MERS-CoV), infectious bronchitis virus (IBV) and porcine epidemic diarrheavirus (PEDV) were simultaneously detected. Hundred microliter of each sample containing 200 ng spike protein was added to the sample pad of the test strip and incubated for 10 min at room temperature. Other respiratory diseases including A/Swine/Guangxi/NN1994/2013 (H1N1), A/Swine/Guangxi/NNXD/2016 (H3N2), A/Duck/Yunnan/YN-9/2016 (H5N6), A/Chicken/Huizhou/HZ-3/2016 (H7N9) and A/Chicken/Guangdong/V/2008 (H9N2) were simultaneously detected. Hundred microliter of each sample containing 10^5^ TCID_50_ allantoic fluid was added to the sample pad of the test strip and incubated for 10 min at room temperature.

### Sensitivity Evaluations of the Strip

Strip sensitivity was determined using a serial diluted of SARS-CoV-2 RBD protein produced in this study (1 mg/mL) and S1 protein (1 mg/mL, Sino Biological Inc.). The protein of SARS-CoV-2 RBD protein produced in this study was diluted 2 times from 4,000 to 15.63 ng/mL with 0.01 M PBS and S1 protein (1 mg/mL, Sino Biological Inc.) was diluted 2 times from 4,000 to 62.5 ng/mL with 0.01 M PBS. Hundred microliter of each sample was added to the sample pad of the test strip and incubated for 10 min at room temperature. Test lines were scanned with TSR3000 Membrane Strip Reader (BioDot, USA) to obtain the relative optical density (ROD). The result was displayed as G/D × area (graphdensity × area)—ROD value, and it was considered positive if it was >10, otherwise it was negative.

### Stability Evaluations of the Strip

These strips were tested to determine their sensitivity in detecting SARS-CoV-2 RBD protein produced in this study upon storage at room temperature for 6 months. The SARS-CoV-2 RBD protein produced in this study was diluted 2 times from 4,000 to 15.63 ng/mL with 0.01 M PBS and 100 μL of the sample was added to the sample pad of the test strip and incubated for 10 min at room temperature.

## Results and Discussion

### Production and Purification of the Recombinant Spike Protein RBD of SARS-CoV-2

**Figure 1:**
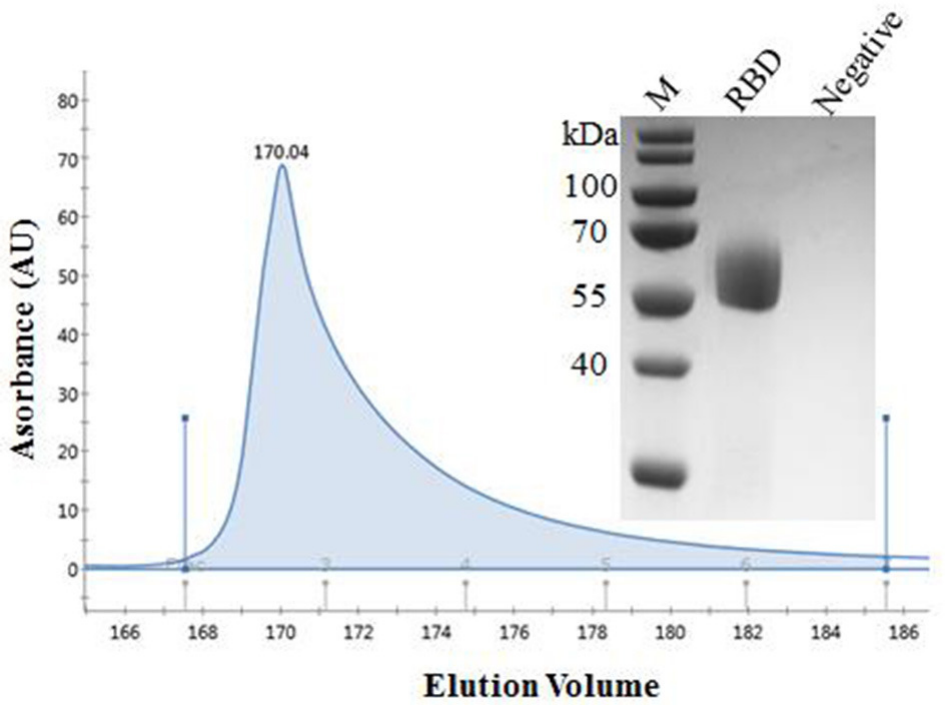
Purification of SARS-CoV-2 RBD protein. Analytical gel filtration profile of SARS-CoV-2 RBD protein with HisTrap^TM^ excel. The 280-nm absorbance curve was shown. SDS-PAGE migration profiles of the sample purified was shown.

The recombinant spike protein RBD of SARS-CoV-2 was produced from the transfected CHO cells. The supernatant was harvested and the secreted proteins were purified. The result of SDS-PAGE showed that the protein was eluted in PBS containing 50 mM imidazole and the purity was >90%, laying the foundation for the next step of mouse immunization (Figure 1). The standard curve was constructed by plotting the absorbance and BSA protein concentration. Finally, the protein concentration of RBD was calculated to be 1 mg/ mL.

### Preparation and Characterization of Monoclonal Antibodies

Before the last immunization, blood was collected from the tail to determine the antibody titer of the mice. The ELISA titers of the mice positive serum ranged from 1: 6,400 to 1: 12,800. Splenocytes from the mouse 2 with the highest titer was fused with Sp2/0 myeloma cells (Table 2). Then sixteen McAbs against spike protein of SARS-CoV-2 were produced in this study and the ELISA titers of the McAbs ranged from 10^−4^ from 10^−6^. The subtype identification results showed that the heavy chain of ten McAbs were IgG1, the heavy chain of three McAbs were IgG2a, the heavy chain of two McAbs were IgG2b, the heavy chain of one McAb was IgM and the light chain were all κ chains (Table 3).

**Table 2:** ELISA titers of immunized mice.

**Mouse label**	**Immune dose**	**ELISA titer**
1	0 μg	0
2	20 μg	1: 12,800
3	50 μg	1: 6,400
4	100 μg	1: 6,400
5	200 μg	1: 6,400

**Table 3:** Biological properties of SARS-CoV-2-specific McAbs generated in this study.

**McAbs**	**Isotype**	**ELISA titer**
1F12	IgG1	10^−5^
2C3	IgG1	10^−6^
5C8	IgG1	10^−4^
5E6	IgG2a	10^−4^
5E11	IgG1	10^−5^
6A5	IgG1	10^−4^
6B7	IgG1	10^−5^
6C7	IgG1	10^−4^
6E5	IgG2b	10^−5^
8G6	IgG2a	10^−6^
7C7	IgG1	10^−4^
7H5	IgG1	10^−5^
8C11	IgG1	10^−4^
9F2	IgG2b	10^−6^
10D3	IgM	10^−4^
12F5	IgG2a	10^−5^

### Double-Antibody Sandwich Dot-Blot

**Figure 2:**
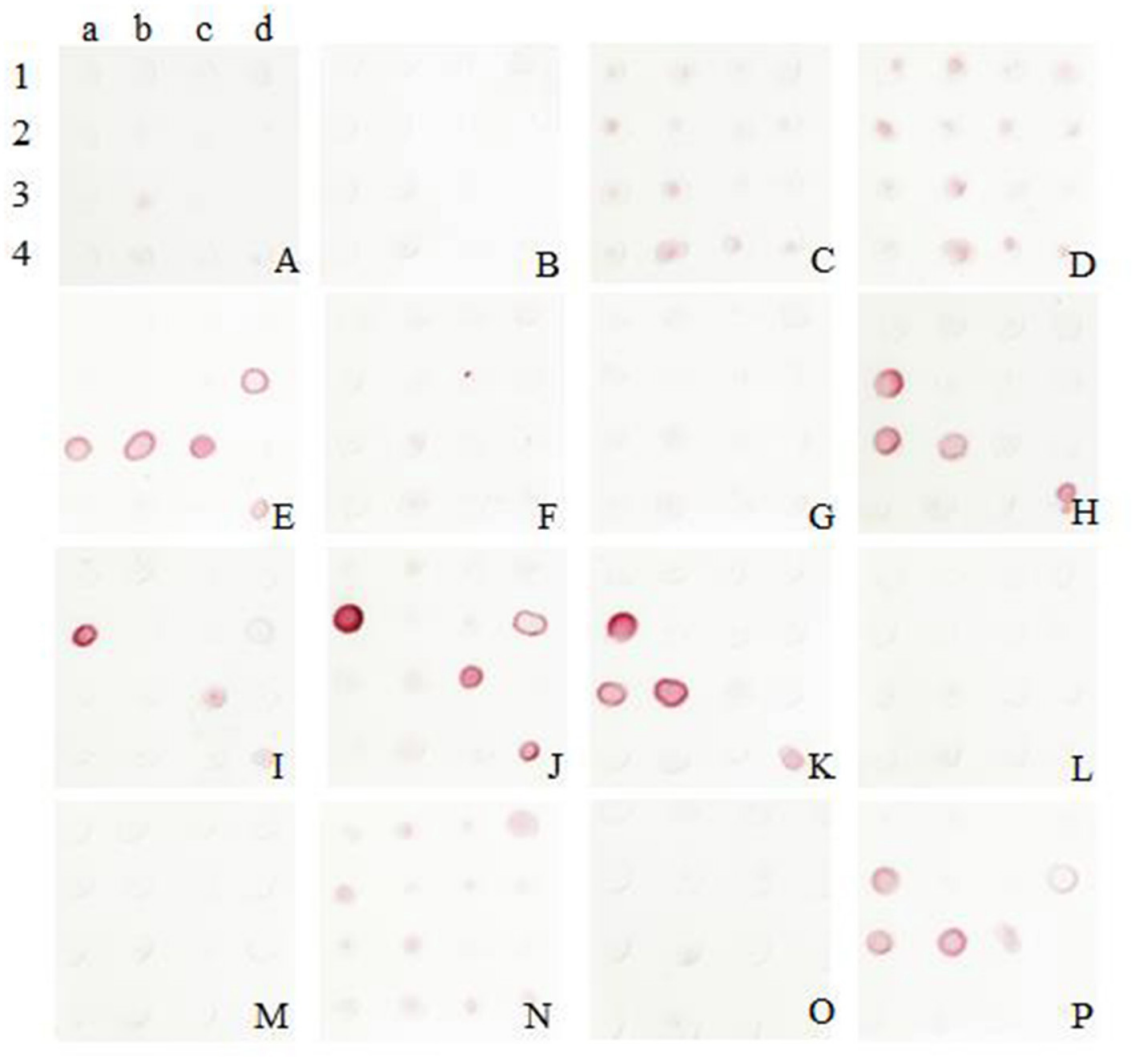
Screening of McAbs for immunochromatographic strip by Sandwich Dot-Blot. 1a−4d: the imprinting distribution of sixteen capture antibodies on 16 nitrocellulose membranes and sample diluted in antigen dilution buffer were added to per membrane; **(A–P)** sixteen colloidal gold conjugated McAbs was added to sixteen membranes, respectively. The pairing of two specific antibodies were selected by observing the color strength of the nitrocellulose membrane.

The colloidal gold was obtained and labeled with sixteen McAbs. After several comparable experiments, the optimum pH was found to be 6, and the optimum labeled McAbs dose was 2 μg/mL. It was validated that the McAb 5E11 used as capture antibody and McAb 8G6 (J2a) used for detection showed the best performance according to the color development in this assay (Figure 2).

### Establishment of a Rapid Detective Immunochromatographic Strip

The colloidal gold conjugation of 8G6 was dispensed on the fiberglass pads as conjugated McAb. The McAb 5E11 was diluted to 0.9 mg/mL in physiological saline and dispensed on the nitrocellulose membrane as the capture test line. Then the SPA was diluted to 0.3 mg/mL in physiological saline as control line. The two specific McAbs of SARS-CoV-2 were detected SARS-CoV-2 by a double antibody sandwich mode.

### Specificity Evaluation of the Strip

A rapid detection strip for the double antibody sandwich mode was established using SARS-CoV-2 specific McAbs 8G6 and 5E11 as conjugation and capture antibodies, respectively.

**Figure 3:**
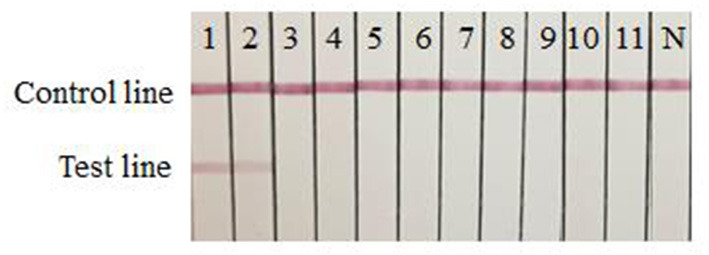
Specificity evaluation of the strip. 1: SARS-CoV-2 RBD protein produced in this study; 2: SARS-CoV-2 S1 protein (Sino Biological Inc.); 3: SARS-CoV S1 protein (Sino Biological Inc.); 4: MERS-CoV S1 protein (Sino Biological Inc.); 5: IBV-S protein (Shandong Lvdu Bio-technique Industry); 6: PEDV-S protein (Shandong Lvdu Bio-technique Industry); 7: A/Swine/Guangxi/NN1994/2013 (H1N1); 8: A/Swine/Guangxi/NNXD/2016 (H3N2); 9: A/Duck/Yunnan/YN-9/2016 (H5N6); 10: A/Chicken/Huizhou/HZ-3/2016 (H7N9); 11: A/Chicken/Guangdong/V/2008 (H9N2); N: PBS negative control.

The specificity test results showed that only SARS-CoV-2 RBD protein produced in this study and S1 protein (Sino Biological Inc.) had two red bands at the T and C lines, SARS-CoV, MERS-CoV, IBV, PEDV and other respiratory diseases had only one red band at the C line (Figure 3), indicating that the rapid detection strip had high specificity for the detection of SARS-CoV-2.

### Sensitivity Evaluation of the Strip

**Figure 4:**
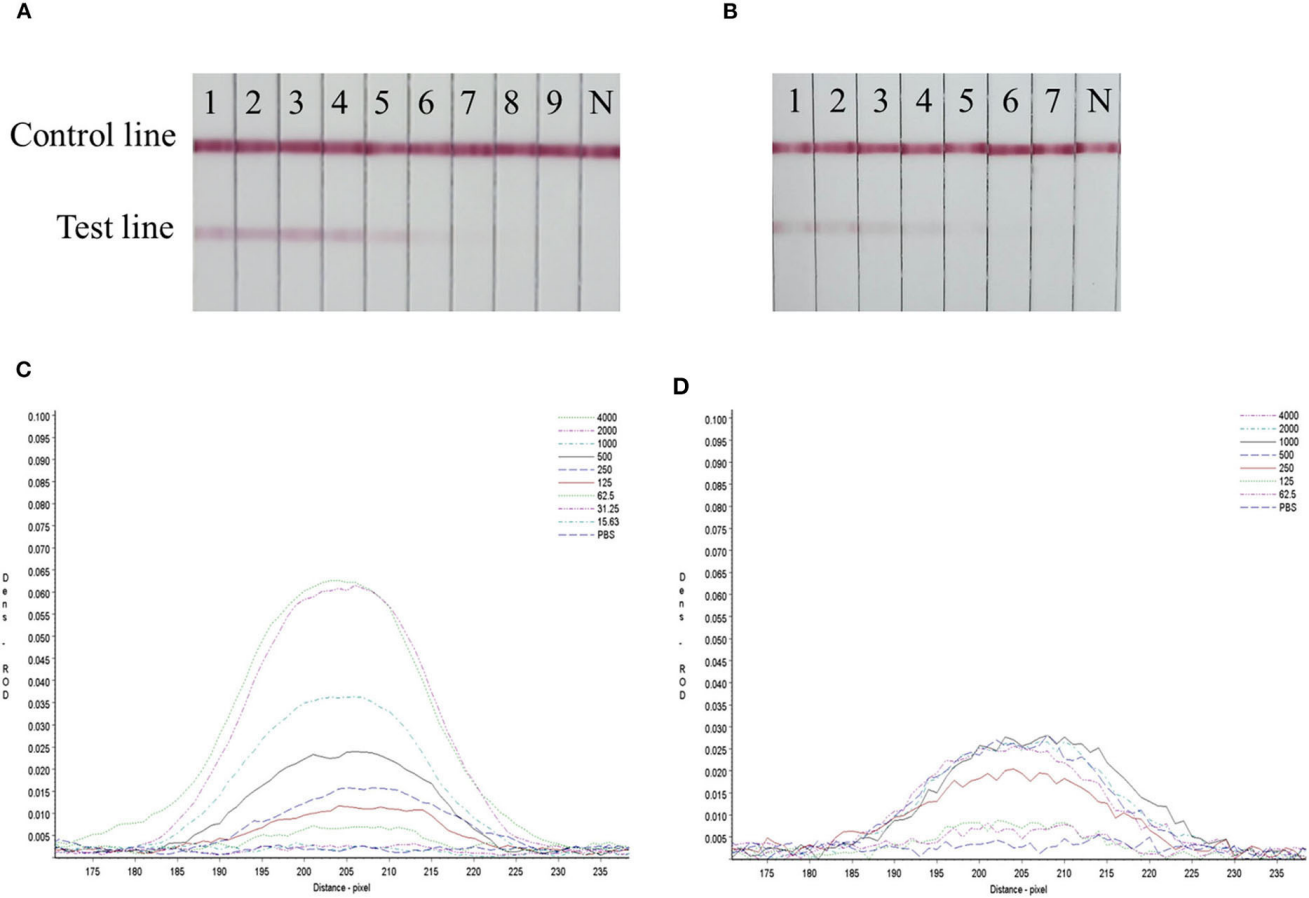
Sensitivity evaluation of the strip. **(A)** SARS-CoV-2 RBD protein produced in this study, 1–11: diluted positive sample ranging from 4,000 to 15.63 ng/mL by two times ratio, N: PBS negative control. **(B)** SARS-CoV-2 S1 protein (Sino Biological Inc.); 1–9: diluted positive sample ranging from 4,000 to 62.5 ng/mL by two times ratio, N: PBS negative control. **(C,D)** The colored membranes of SARS-CoV-2 RBD protein produced in this study and SARS-CoV-2 S1 protein (Sino Biological Inc.) were screened under a TSR-3000 Reader, and relative optical density (ROD) values were analyzed by AIS software.

Serial dilutions of the SARS-CoV-2 RBD protein produced in this study ranging from 4,000 to 15.63 ng/mL and SARS-CoV-2 S1 protein (Sino Biological company) ranging from 4,000 to 62.5 ng/mL were used to determine the sensitivity of the strip. The G/D × area-ROD values reduced as the protein concentration in the samples reduced, so the results showed that the detection limit of RBD was 62.5 ng/mL and the S1 protein was 250 ng/mL (Figure 4 and Table 4).

**Table 4:** Sensitivity evaluation of the rapid detective assay.

**RBD concentration (ng /mL)**	**G/D × area-ROD (pixel)**	**S1 protein concentration (ng /mL)**	**G/D × area-ROD (pixel)**
4,000	100.1451	4,000	44.9483
2,000	93.7376	2,000	41.9143
1,000	55.2415	1,000	32.4942
500	36.5036	500	11.9429
250	24.2338	250	12.3122
125	18.5939	125	6.8560
62.5	11.8163	62.5	3.9135
31.25	5.3165	31.25	NT
15.63	5.3438	15.63	NT
PBS	3.5125	PBS	0

*G/D × area – ROD (pixel) = graph density × area-relative optical density. NT, not tested*.

### Stability Evaluation of the Strip

**Figure 5:**
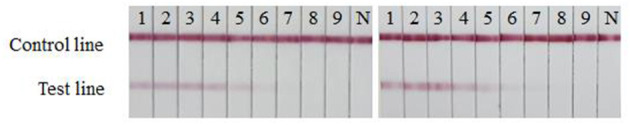
Stability evaluation of the strip. The sensitivities of fresh strips **(left)** and strips after 6 months of storage **(right)** were determined. 1–9: The SARS-CoV-2 RBD protein produced in this study diluted ranging from 4,000 to 62.5 ng/mL by two times ratio; N: PBS negative control.

The strips still had the same detection limit (62.5 ng/mL) for SARS-CoV-2 RBD protein produced in this study as freshly produced strips after 6 months of storage, indicating that the gold immunochromatographic strip had good stability (Figure 5).

## Discussion

Since the outbreak of the COVID-19 pandemic in December 2019, we have been committed to the research and development of the SARS-CoV-2 subunit vaccine. Up to now, the vaccine we researched is in the final stage of preclinical testing. The development of a highly expressed and correctly folded spike protein of SARS-CoV-2 is the basis of subunit vaccines. Gao et al. presented a coronavirus immunogen containing the virus spike protein RBD that expressed in CHO cells (23). Daniel et al. proposed the concept of RBD and expressed spike protein RBD in Expi293F cells (Gibco #A14527) (24, 25). Many scholars choose eukaryotic systems to express proteins because eukaryotic systems have many irreplaceable advantages of other protein expression systems. The advantage of the eukaryotic expression system is that it can induce high-efficiency expression, fold the expressed protein correctly, and carry out complex glycosylation modification. The protein activity is close to that of the natural protein, without the need to remove endotoxin. Therefore, we expressed spike protein RBD of SARS-CoV-2 in CHO cells. The spike protein RBD transient expressed in this study reached g/L level expression and >95% purity, laying the foundation for the next step of mouse immunization and subunit vaccine development.

A total of 16 McAbs were developed, two McAbs 8G6 and 12F5 were selected as conjugation and capture antibodies for the strip by sandwich Dot-blot. To avoid low affinity and possible cross-reactivity, McAbs were screened against different epitopes of the SARS-CoV-2 antigen by the sandwich method. The remaining 14 McAbs can be not only used in vaccine production and basic research of immunology, but also in pathogen detection, antigen purification, and disease diagnosis and prevention (26). The biological characteristics of monoclonal antibodies are a key factor in determining the performance of colloidal gold immunochromatography strip. Due to the unstable ascites of different batches, we prepared a large amount of ascites and purified it in aliquots for storage to prevent repeated exploration of the best conditions in order to ensure the availability of subsequent experimental data.

Colloidal gold immunochromatography is a novel rapid immunological assay developed in the 1980s (27). Colloidal gold immunochromatography has always been a hot research topic (28–30). Zhang et al. (31) developed a rapid diagnostic test strip for detecting infectious bursal disease virus (IBDV). The colloidal gold immunochromatography technology is convenient to apply, and the results show rapid and intuitive, and rapid application in the field of animal medicine. The rapid spread of SARS-CoV-2 around the world has resulted in a large number of confirmed and fatal cases and may increase the risk of human infections. Prompt identification and isolation of SARS-CoV-2 patients would help prevent the spread of SARS-CoV-2 and potentially a pandemic. Under extreme conditions, such as in remote countries and regions with backward equipment and technology, in order to control and manage the epidemic in time, test strips can also be used as a diagnostic tool. Spike protein can represent a positive for the SARS-CoV-2 to a certain extent. When the test paper is used as a diagnostic tool, the sensitivity may not be comparable with molecular detection such as RT-PCR. The sensitivity of the test strip can be tried to improve through new nanomaterial labels and biosensors in our follow-up experiments. At the same time, the immunochromatographic strip developed in this study plays an important role in the production of subunit vaccines. For example, in the protein production process, the immunochromatographic strip developed in this study with a detection limit of 62.5 ng/mL can detect the protein content in the cell culture flask, preventing the accumulation of protease in the cell culture for too long from affecting the protein yield. During the protein purification process, the immunochromatographic strip can detect the protein content in the collection tube, so as not to stop the purification too early or too late and cause the protein yield to decrease. The immunochromatographic strip can also be used to monitor the spike protein content and vaccine stability of the SARS-CoV-2 vaccine on the market in the future.

## Data Availability Statement

The raw data supporting the conclusions of this article will be made available by the authors, without undue reservation.

## Ethics Statement

The animal study was reviewed and approved by Key Laboratory of Animal Immunology, Henan Academy of Agricultural Sciences.

## Author Contributions

GL, AW, YC, YD, XW, and GZ designed the research and analyzed the data. PD, RJ, and YW provided resources. GL, AW, GZ, and XW performed the experiments and wrote the manuscript. All authors contributed to the article and approved the submitted version.

## Conflict of Interest

The authors declare that the research was conducted in the absence of any commercial or financial relationships that could be construed as a potential conflict of interest.
